# A Scoping Review of Gaps Identified by Primary Care Providers in Caring for Patients with Chronic Noncancer Pain

**DOI:** 10.1080/24740527.2022.2145940

**Published:** 2023-02-17

**Authors:** Virginia McEwen, Mihal (Michelle) Esterlis, R. Gianni Lorello, Abhimanyu Sud, F. Marina Englesakis, Anuj Bhatia

**Affiliations:** a Chronic Pain Management Program, St. Joseph’s Care Group, Thunder Bay, ON, Canada; b Interventional Pain Service, Thunder Bay Regional Health Sciences Centre, Thunder Bay, ON, Canada; c Clinical Sciences Division, Northern Ontario School of Medicine University, Thunder Bay, ON, Canada; d Department of Nursing, Ryerson University, Toronto, ON, Canada; e Department of Anesthesiology and Pain Medicine, University of Toronto, Toronto, ON, Canada; f Department of Anesthesia and Pain Medicine, University Health Network - Toronto Western Hospital, Toronto, ON, Canada; g Women’s College Research Institute, Women’s College Hospital, Toronto, ON, Canada; h Wilson Centre, Toronto General Hospital, University of Toronto, Toronto, ON, Canada; i Department of Family and Community Medicine, University of Toronto, Toronto, ON, Canada; j Humber River Hospital, Toronto, ON, Canada; k Institute of Health Policy, Management and Evaluation, University of Toronto, Toronto, ON, Canada; l Library & Information Services, University Health Network, Toronto, ON, Canada

**Keywords:** scoping review, chronic noncancer pain, primary care providers, medical education

## Abstract

**Introduction/Aim:**

Primary care providers (PCPs), who provide the bulk of care for patients with chronic noncancer pain (CNCP), often report knowledge gaps, limited resources, and difficult patient encounters while managing chronic pain. This scoping review seeks to evaluate gaps identified by PCPs in providing care to patients with chronic pain.

**Methods:**

The Arksey and O’Malley framework was used for this scoping review. A broad literature search was conducted for relevant articles on gaps in knowledge and skills of PCPs and in their health care environment for managing chronic pain, with multiple search term derivatives for concepts of interest. Articles from the initial search were screened for relevance, yielding 31 studies. Inductive and deductive thematic analysis was adopted.

**Results:**

The studies included in this review reflected a variety of study designs, settings, and methods. However, consistent themes emerged with respect to gaps in knowledge and skills for assessment, diagnosis, treatment, and interprofessional roles in chronic pain, as well as broader systemic issues including attitudes toward CNCP. A general lack of confidence in tapering high dose or ineffective opioid regimes, professional isolation, challenges in managing patients with CNCP with complex needs, and limited access to pain specialists were reported by PCPs.

**Discussion/Conclusions:**

This scoping review revealed common elements across the selected studies that will be useful in guiding creation of targeted supports for PCPs to manage CNCP. This review also yielded insights for pain clinicians at tertiary centers for supporting their PCP colleagues as well as systemic reforms required to support patients with CNCP.

## RÉSUMÉ

**Introduction/Objectif:** Les prestataires de soins primaires, qui fournissent la majeure partie des soins aux patients souffrant de douleur chronique non cancéreuse, font souvent état de lacunes dans leurs connaissances, de ressources limitées et de rencontres difficiles avec les patients dans le cadre de la prise en charge de la douleur chronique. Cet examen de la portée vise à évaluer les lacunes identifiées par les prestataires de soins primaires dans la prestation de soins aux patients souffrant de douleur chronique.

**Méthodes:** Le cadre d’Arksey et O’Malley a été utilisé pour cet examen de la portée. Une vaste recherche documentaire a été menée pour trouver des articles pertinents sur les lacunes dans les connaissances et les compétences des prestataires de soins primaires et dans leur environnement de soins de santé pour la prise en charge de la douleur chronique, avec de multiples dérivés de termes de recherche pour les concepts d’intérêt. Les articles de la recherche initiale ont été examinés pour leur pertinence, ce qui a donné 31 études. L’analyse thématique inductive et déductive a été adoptée.

**Résultats:** Les études incluses dans cette revue reflétaient une variété de devis, de milieux et de méthodes d’étude. Cependant, des thèmes récurrents ont émergé en ce qui concerne les lacunes dans les connaissances et les compétences concernant l’évaluation, le diagnostic, le traitement et les rôles interprofessionnels dans la douleur chronique, ainsi que des problèmes systémiques plus larges, y compris les attitudes à l’égard de la douleur chronique non cancéreuse. Un manque général de confiance dans les traitements opioïdes à dose élevée ou inefficaces, l’isolement professionnel, les difficultés de prise en charge des patients atteints de douleur chronique non cancéreuse ayant des besoins complexes et l’accès limité aux spécialistes de la douleur ont été signalés par les prestataires de soins primaires.

**Discussion/Conclusions:** Cet examen de la portée a révélé des éléments communs aux études sélectionnées qui seront utiles pour guider la création de soutiens ciblés pour les prestataires de soins primaires dans le cadre de la prise en charge de la douleur chronique non cancéreuse. Cette revue a également fourni des informations aux cliniciens de la douleur des centres tertiaires pour soutenir leurs collègues prestataires de soins primaires ainsi que les réformes systémiques nécessaires pour soutenir les patients atteints de douleur chronique non cancéreuse.

## Introduction

### Significance of Primary Care in Treating Chronic Pain

Family physicians, general practitioners, nurse practitioners (NPs), clinical nurse specialists, and physician assistants are all considered primary care providers (PCPs)^1^ and are often the first point of contact for the majority of patients with chronic noncancer pain (CNCP) in Canada. CNCP is generally defined as pain persisting beyond the normally expected time for healing, with 3 months as the typical threshold for a diagnosis of chronic pain.^2^ Given that nearly one in five individuals in Canada experience CNCP, treating pain is a significant proportion of the care delivered by PCPs.^3–9^ These providers offer an early point of contact with the health care system for patients with chronic pain; they provide comprehensive care, and they can also identify patients who are in need of specialist care.^10^ With the increasing complexity of primary care and expectations of stronger opioid stewardship since the 2017 Canadian Guidelines for Opioid Therapy and Chronic Noncancer Pain^11^ publication, a need to assess the challenges faced by PCPs in providing the bulk of care to patients living with CNCP exists.

### Background

PCPs often receive inadequate education and training in treating and managing chronic pain.^12–14^ Even those PCPs who receive exposure to diagnosing and treating chronic pain may find it difficult to translate this knowledge into optimal outcomes for a multitude of reasons, including lack of reliable and objective measures of pain, concerns about adverse effects of analgesic treatments, and limitations in contact time with patients.^15–17^ It is therefore not surprising that individuals experiencing CNCP often feel that their complaints are addressed with skepticism, lack of understanding, rejection, belittlement, or blame or labeled with diagnoses such as somatic symptom disorder that implies that their pain is a psychological construct.^18^ Health care professionals struggle to understand the subjective nature of pain, often finding difficulty in correlating pain complaints with objective findings on physical examination and investigations, and have difficulty empathizing with patients’ suffering when secondary gain may be a consideration.^19^ Further, fears of contributing to opioid dependence and causing harm to patients^19^ are relevant concerns in the management of CNPC. Prescribing of opioids in high doses, a practice more prevalent in the past that continues to cast a shadow in current management of CNCP, is now known to carry significant risks and complications and adds complexity in CNCP care.^20^

PCPs are often expected to provide comprehensive CNCP care regardless of whether their training has equipped them with the tools to do so.^21^ This can lead to professional frustration and less-than-ideal outcomes for patients with CNCP.^22^ Medical and nursing schools historically have not allocated significant time to teach assessment and management of pain, which has resulted in suboptimal knowledge and skill levels of PCPs treating patients with CNCP, a problem that potentially compromises patient care.^21^

### Rationale for This Review

Though the Pain Medicine specialty training program of the Royal College of Physicians and Surgeons of Canada^23^ is a welcomed new addition to the postgraduate training landscape, access to advanced pain specialists historically has been limited, and the bulk of chronic pain management is delivered by PCPs.^24^ There is a current need to understand the challenges faced by PCPs after the recent substantive shift in opioid prescribing practices, the concurrent growth of pain medicine, and the present impetus to advance care for CNCP in Canada with the recent release of the Canadian Pain Task Force recommendations.^25^

### Aims

The broad aims of this scoping review were to provide an overview of the perspectives of Canadian PCPs in managing CNCP, identify gaps in the existing literature, and explore the potential to use the results of this review in the future to guide continuing medical education curricula and other supports for PCPs.^26,27^

We considered a scoping review of literature followed by a discussion of the available evidence as appropriate for this topic. Scoping reviews seek to map emerging literature in terms of its volume, nature, and characteristics. In contrast to systematic reviews, scoping reviews do not answer a focused research question.^26^ Scoping reviews further differ from systematic reviews in that they address broader topics in which different study designs may be applicable, and thus the heterogeneous nature of available literature on this subject justifies this approach of synthesizing evidence.^28^ Furthermore, though a scoping review has been done on PCP opioid prescribing safety measures,^29^ also covered in part by various publications at different time points and in multiple regions of Canada, this subject has not yet been extensively reviewed. Our review is therefore one of the first comprehensive syntheses to assimilate evidence on primary care perspectives on the management of CNCP pain.

## Materials and Methods

**Figure 1.**
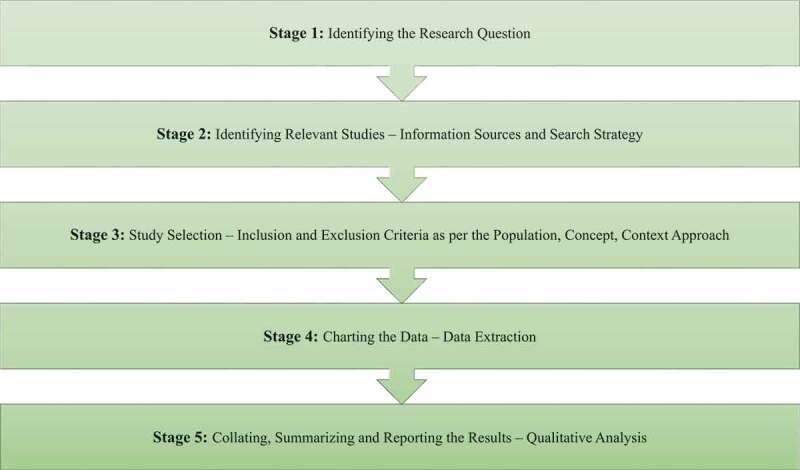
Flow diagram of methodology process and analysis.

This scoping review was informed by the framework proposed by Arksey and O’Malley,^26^ which was updated by Levac and colleagues.^27^ We conducted this review as per the five stages of the Arksey and O’Malley framework: identifying the research question (stage 1), identifying relevant studies (stage 2), study selection (stage 3), charting the data (stage 4), and collating, summarizing, and reporting the results (stage 5; Figure 1).^26^ The reporting of this scoping review followed the Preferred Reporting Items for Scoping Reviews guidelines.^30^ The focus of this review was on comprehensiveness rather than depth, as recommended for scoping reviews.^26^ This scoping review relies on published research in the public domain and therefore did not require research ethics board review; because it also does not include living human participants, this exempted the need for informed consent.

### Stage 1: Identifying the Research Question

The primary objective of this review was to identify gaps reported by PCPs in managing CNCP, which included knowledge and skills but also systemic barriers and overarching attitudes and beliefs around CNCP. The secondary objective was to identify approaches reported for addressing these gaps with knowledge translation strategies, approaches to change attitudes and beliefs about people living with CNCP, and examining ways in which tertiary pain centers can optimize shared care of patients with PCPs.

### Stage 2: Identifying Relevant Studies—Information Sources and Search Strategy

The information specialist (M.F.E.) conducted a systematic search of the following databases from their inception via the Ovid platform: MEDLINE ALL (1946–), Embase Classic/Embase (1947–), Cochrane Central Registry of Controlled Trials (1995–), and Cochrane Database of Systematic Reviews (2005–). CINAHL Complete (via the EbscoHOST platform, 1982–) was also searched. The trial registries ClinicalTrials.Gov (National Institutes of Health) and World Health Organization International Clinical Trials Platform were also searched. Dissertations were sought using the ProQuest Digital Dissertations International database. Lastly, books or book chapters were sought using the UHN’s Summon OneSearch discovery service. All databases and trial registries were searched on the same day, January 2, 2020. Update searching over all databases and registries was conducted on May 12, 2021.

The search process followed the Cochrane Handbook^31^ and the Cochrane Methodological Expectations of Cochrane Intervention Reviews^32^ for conducting the search. The PRESS guideline for peer-reviewing the search strategies,^33^ drawing upon the PRESS 2015 Guideline Evidence-Based Checklist, was used to avoid potential search errors.

To develop comprehensive search strategies, preliminary searches were conducted, and full-text literature was mined for potential keywords and appropriate controlled vocabulary terms (such as Medical Subject Headings for MEDLINE and EMTREE descriptors for Embase). The Yale MeSH Analyzer^34^ was used to facilitate the MeSH and text word analysis, using target citations provided by the team.

The search strategy concept blocks were built on the following topics: (primary care physicians or related terms) AND (chronic pain or related terms) AND (questionnaires or surveys or related terms) AND (Canada, including all provinces and territories) using both controlled vocabularies and text word searching for each component. Searches were limited to English language.

The Ovid MEDLINE ALL search strategy is provided in Appendix A.

**Table:** 

No.	Searches
1	Primary Health Care/
2	Physicians, Primary Care/
3	(primary adj2 care).mp.
4	primary healthcare.mp.
5	exp Physicians, Family/
6	exp Family Practice/
7	exp Community medicine/
8	exp Physician’s Practice Patterns/ and (family or general pract* or primary care or primary health*).mp.
9	exp Regional medical programs/
10	exp homes for the aged/
11	house calls/
12	house call:.mp.
13	housecall:.mp.
14	house visit*.mp.
15	home visit*.mp.
16	Rural Health Services/
17	Hospitals, Rural/
18	Rural Health/
19	(free-standing adj2 clinic?).mp.
20	(free-standing adj2 facilit:).mp.
21	community health centers/
22	child guidance clinics/
23	maternal-child health centers/
24	family doctor?.mp.
25	“family and community medicine.”mp.
26	family medicine.mp.
27	family physician?.mp.
28	family practitioner?.mp.
29	general practice?.mp.
30	general practise?.mp.
31	general practitioner?.mp.
32	(personal adj1 (doctor? or physician? or practitioner?)).mp.
33	primary care provider?.mp.
34	primary health care.mp.
35	primary healthcare.mp.
36	primary health* provider?.mp.
37	pcp staff.mp.
38	community physician?.mp.
39	(resident? adj25 (physician? or doctor? or clinician?)).mp.
40	(intern? adj25 (physician? or doctor? or clinician?)).mp.
41	(trainee? adj50 (physician? or doctor? or clinician?)).mp.
42	or/1-41 [Primary Health Care or General Practice or Family Practice]
43	Chronic Pain/ [MeSH term since 2012]
44	Causalgia/
45	exp Arthralgia/
46	exp Back Pain/
47	exp Central Nervous System/ and exp *”Wounds and Injuries”/
48	exp Central Nervous System/ and exp Pain/
49	exp Chronic Illness/ and exp Pain/ [Historical search for chronic pain]
50	exp Complex Regional Pain Syndromes/
51	exp Diabetic Neuropathies/
52	exp Headache/
53	exp Headache Disorders/
54	exp Herpes Zoster/
55	exp Hyperalgesia/
56	exp Mononeuropathies/
57	exp Nerve Compression Syndromes/
58	exp Neuralgia/
59	exp Neurons, Afferent/
60	exp Nociceptors/
61	exp Palliative Care/
62	exp Pelvic Pain/
63	exp Peripheral Nervous System/ and exp *”Wounds and Injuries”/
64	exp Peripheral Nervous System/ and exp Pain/
65	exp Polyneuropathies/
66	Glossalgia/
67	Mastodynia/
68	Metatarsalgia/
69	Piriformis Muscle Syndrome/
70	Reflex Sympathetic Dystrophy/
71	(afferent adj2 neuron?).mp,kw.
72	(back? adj2 pain*).mp,kw.
73	(cancer* adj3 pain*).mp.
74	(chronic* adj2 headache?).mp,kw.
75	(chronic* adj2 head-ache?).mp,kw.
76	(chronic* adj2 migrain*).mp,kw.
77	(chronic* adj3 pain*).mp,kw.
78	(deafferentation adj2 pain*).mp,kw.
79	(deafferentation adj2 pain*).mp,kw.
80	(dysa?sthetic adj2 pain*).mp,kw.
81	(maladapt* adj2 pain*).mp,kw.
82	(mal-adapt* adj2 pain*).mp,kw.
83	(mononeurit* adj1 multiple*).mp,kw.
84	(mono-neurit* adj1 multiple*).mp,kw.
85	(nerve? adj12 pals???).mp,kw.
86	(nerve? adj2 damag*).mp,kw.
87	(nerve? adj2 injur*).mp,kw.
88	(nerve? adj2 injur*).mp,kw.
89	(nerve? adj2 sensitiv*).mp,kw.
90	(nerve? adj3 entrap*).mp,kw.
91	(neural adj2 damag*).mp,kw.
92	(neural adj2 injur*).mp,kw.
93	(neural adj3 entrap*).mp,kw.
94	(neural adj3 sensitiv*).mp,kw.
95	(neuro* adj2 pain*).mp,kw.
96	(neuro* adj2 pain*).mp,kw.
97	(neuro* adj2 sensitiv*).mp,kw.
98	(neuropath* adj2 pain*).mp,kw.
99	(pain adj2 low?? adj2 back?).mp,kw.
100	(pain adj5 multiple scleros*).mp,kw.
101	(pain or pains or pained or painful*).mp,kw.
102	(pain* adj3 syndrom*).mp,kw.
103	(pelvic adj5 pain*).mp,kw.
104	(peripheral* adj1 mononeurit*).mp,kw.
105	(peripheral* adj1 mono-neurit*).mp,kw.
106	(peripheral* adj1 neurit*).mp,kw.
107	(peripheral* adj1 polyneurit*).mp,kw.
108	(peripheral* adj1 poly-neurit*).mp,kw.
109	allodynia*.mp,kw.
110	arthralgi*.mp,kw.
111	causalgi*.mp,kw.
112	cephalalgi*.mp,kw.
113	cephalgi*.mp,kw.
114	chronic noncancer* pain?.mp,kw.
115	chronic non-cancer* pain?.mp,kw.
116	chronic nonmalignan* pain?.mp,kw.
117	chronic non-malignan* pain?.mp,kw.
118	colic.mp,kw.
119	dysaesthesi*.mp,kw.
120	dysesthesi*.mp,kw.
121	dysmenorrhea*.mp,kw.
122	dysmenorrhoea*.mp,kw.
123	earache?.mp,kw.
124	ear-ache?.mp,kw.
125	failed back?.mp,kw.
126	glossalgi*.mp,kw.
127	headach???.mp.
128	head-ach???.mp.
129	Herpes Zoster.mp,kw.
130	hyper?esthesi*.mp,kw.
131	hyperalges*.mp,kw.
132	hyperpathi*.mp,kw.
133	hypo?esthesi*.mp,kw.
134	mastodyni*.mp,kw.
135	metatarsalgi*.mp,kw.
136	migrain*.mp,kw.
137	mononeuropath???.mp,kw.
138	mono-neuropath???.mp,kw.
139	neuralgi*.mp,kw.
140	neuropath*.mp,kw.
141	neuropathic.mp,kw.
142	neuropathies.mp,kw.
143	neuropathy.mp,kw.
144	nocicept*.mp,kw.
145	palliat*.mp,kw.
146	paraesthesi*.mp,kw.
147	paresthesi*.mp,kw.
148	phantom limb?.mp,kw.
149	piriformis muscle syndrome?.mp,kw.
150	polyneuropath???.mp,kw.
151	poly-neuropath???.mp,kw.
152	reflex sympathetic dystroph*.mp,kw.
153	sciatic??.mp,kw.
154	shingles.mp,kw.
155	somatosensory.mp,kw.
156	toothache?.mp,kw.
157	tooth-ache?.mp,kw.
158	or/43-157 [Chronic Pain]
159	42 and 158 [PCP + Chronic Pain]
160	*Data Collection/
161	“Surveys and Questionnaires”/
162	Focus Groups/
163	Health Knowledge, Attitudes, Practice/
164	“Health Services Needs and Demand”/
165	Interviews As Topic/
166	Narration/
167	Needs Assessment/
168	Qualitative Research/
169	content analys*.mp.
170	(data adj1 collect*).mp.
171	(discussion or discussions).mp.
172	focus group*.mp,kw.
173	interview*.mp,kw.
174	(need? adj3 assess*).mp.
175	opinion?.mp.
176	poll?.mp,kw.
177	qualitative*.mp.
178	question???.mp.
179	questionnaire?.mp,kw.
180	self-report*.mp.
181	survey*.mp,kw.
182	(theme or themes).mp.
183	or/160-182 [Questionnaires or Surveys and related terms]
184	159 and 183 [PCP + Chronic Pain + Questionnaires]
185	exp Canada/
186	Canada.af.
187	Canada.in.
188	canadian?.af.
189	british columbia.mp.
190	alberta.mp.
191	saskatchewan.mp.
192	manitoba.mp.
193	ontario.mp.
194	quebec.mp.
195	new Brunswick.mp.
196	prince edward island.mp.
197	nova scotia.mp.
198	newfoundland.mp.
199	northwest territories.mp.
200	yukon.mp.
201	nunavut.mp.
202	or/185-201 [Canada]
203	184 and 202 [PCP + Chronic Pain + Questionnaires + Canada]
204	limit 203 to english language
205	remove duplicates from 204
206	limit 205 to yr = “2020 -Current”

Supplemental Google Scholar searching was conducted by other members of the team, starting with the following search string: (“primary care physicians” OR “primary care doctors” OR “family physicians” OR “family doctors”) AND “chronic pain” AND (questionnaire OR survey) AND Canada. The first 200 resulting citations from Google Scholar were reviewed.

Lastly, team members also hand-searched reference lists of selected articles to identify additional potentially relevant articles.

### Stage 3: Study Selection—Inclusion and Exclusion Criteria

A population, concept, context approach was followed for this scoping review.^35^ The population of interest included Canadian PCPs including family physicians, general practitioners, nurse practitioners, and their medical learners. Publications with allied health professionals and other medical specialties were included if a participant cohort was clearly identified as PCPs. Medical learners were included as a population that could provide insights into the base education of chronic pain care that PCPs then eventually rely upon in the absence of dedicated postgraduate training. We focused on studies only in the Canadian context given important differences between countries in health care systems, including at primary and tertiary levels, and in pain education curricula between Canada and other jurisdictions. The concepts of interest were gaps in the PCPs’ knowledge and skills, barriers in addressing CNCP, and/or reported strategies for improving CNCP management. The context was patients with CNCP including chronic pain in cancer survivors; studies on acute pain, cancer pain, and palliative care were excluded. Because the goal was foremost to understand the gaps and barriers experienced by PCPs, articles focused on patient perspectives were not included in this article, although these would be highly valuable for subsequent consideration as a separate review.

Eligible study designs were primary empirical studies, including qualitative studies, case reports, case series, observational studies (prospective, retrospective, and using questionnaires or surveys), randomized controlled trials, systematic reviews, and conference abstracts. Editorials and opinion pieces were not included.

Abstracts identified within each search were imported into Rayyan, an evidence synthesis data management software, and duplicates were removed. Articles were screened independently by two reviewers (M.E. and V.M.), who reviewed titles and abstracts for relevance and eligibility. A subsequent full-text review of each article that passed the initial screening was completed independently and in duplicate, and conflicts were resolved through discussion, consensus, and input from the senior author (A.B.).

### Stage 4: Charting the Data—Data Extraction

Data extraction tables were constructed and pilot-tested prior to use. Comprehensive extraction of the data into Table 1 was performed and the accuracy of extracted data was verified by the first two authors (V.M., M.E.) independently. The data extracted and summarized in the table included study characteristics such as author, year, study type, number of participants, the setting/research theme, a summary of methods, and relevant results.

**Table 1.** A summary of studies on PCPs’ (family physicians, general practitioners, nurse practitioners, and medical learners) perceptions of gaps in treating chronic pain included in the review.

First author, year Study type, Number of participants	Setting/Research theme	Methods	Results
Allen 2011; Pre- and post-intervention survey; 13 physicians, 15 dentists, 26 pharmacists	Interprofessional education (IPE) workshop in Cape Breton, Nova Scotia	Workshop with pre and post surveys evaluating impact of IPE for PCPs to increase self-efficacy in managing patients with CNCP, enhance interprofessional communication, use of local resources, and improve prescribing practices of opioids	Self-efficacy scores were significantly higher after the workshop
			75% of 3-month post workshop respondents reported greater involvement of other health professionals in managing CNCP
			50% of 3-month post-workshop participants reported using local resources with concerns around opioid misuse and 25% reported facilitating contact with addiction services
Allen 2013; Cross-sectional survey; 710 respondents	Canadian FPs’ management of CNCP	Determining FPs’ practices and knowledge in prescribing opioids prior to the release of the 2010 Canadian Guidelines on use of opioids for CNCP	Most FPs practiced in alignment with guidelines in monitoring for adverse events, aberrant behaviour, and advising caution with driving
			FPs did not discontinue opioids even when ineffective
			FPs unaware of ‘watchful dose’ of opioids
			Gaps included access to pharmacy registries in some provinces, and access to pain specialists or addiction services
Baer 2010; Cross-sectional survey; 41 physicians with data on 736 chronic pain patients	Primary care physicians in Southern Ontario	Initial detailed questionnaires were collected to profile usual chronic pain management practices followed by an electronic audit of pain management practices using personal digital assistant technology	Chronic pain patients require considerably more time than patients with other ailments
			PCPs reported needing to make compromises with respect to optimal care of chronic pain
			Barriers included costs, adequate dosing of pain medications, and lack of access to multidisciplinary treatment resources
Baer 2012; Cross-sectional survey; 30 primary care physicians	Canadian primary care physicians	Survey questionnaire of primary care physicians pain management practices compare perceptions of pain patterns versus actual patient results, assess adherence to 2010 recommendations from the Canadian Guideline for Safe and Effective Use of Opioids for CNCP, and document factors influencing physician treatment recommendations	Gaps noted in pain management practices included:
			Non-universal use of validated pain scales despite guideline recommendations
			Underestimation of mixed pain despite common use of multimodal analgesia
			Limited use of non-pharmacological treatments despite patients having private coverage for these treatments
Busse 2020; Cross-sectional survey; 1128 physicians (463 family MDs)	Adherence to/altered practices in response to 2017 Canadian guideline for opioids in CNCP	28-item questionnaire Likert scale question and with opportunity for open-ended responses to some items	Respondents acknowledged resistance by patients, financial barriers to nonpharmacological treatment, lack of availability of nonpharmacological treatment as implementation barriers
			Many respondents noted inadequate time to manage complex cases, lack of access to addiction medicine, and unrealistic to taper some high-opioid dose legacy patients
			Respondents reported increased coverage for chronic pain treatment options by insurers, greater availability to chronic pain treatment services would facilitate guideline implementation
			Suggested CME: managing challenging patients on chronic opioids, instruction on nonopioid options for managing CNCP
			Desired formats for CME were small groups, online courses, or archived videos
Carlin 2017; Qualitative descriptive study; 20 participants	ECHO (Ontario Chronic Pain/Opioid Stewardship) program for managing chronic pain in primary care: aims to increase capacity in managing complex cases in areas with poor access to specialists	Focus group interviews regarding the ECHO model: A tele-mentoring intervention for PCP to enhance pain management skills, combining a didactic presentation by an expert from a “hub” of specialists to PCPs in community and allows a PCP to present a challenging case and receive multidisciplinary guidance on further assessment/management	Participants learned about alternatives to opioid prescribing
			Participants acknowledged challenges in accessing non-pharmacological interventions
			Participants appreciated the support in managing complex patients and they felt less isolated
			Physicians commented they had received little training on pain management and opioid prescribing prior to ECHO
Chow 2017; Needs assessment survey; 162 respondents	Pain management by Canadian FPs for cancer survivors	Determining FPs’ needs in treating chronic pain in cancer-survivors	Most FP reported a lack of knowledge in the management of pain in cancer survivors but were keen to receive medical education on treatment options and clinical practice guidelines Deficits included access to specialists and to non-pharmacology interventions (ex. CBT), cost/coverage of medications, concerns about opioid misuse, knowledge of pharmacotherapy options and stepwise approach to pain management, and lack of time for assessing and treating patients
Clark 2015; Randomized experimental design; 78 PCPs Desveaux 2019; Qualitative study; 22 participants	Telephone consultations with PCPs who referred patients to pain clinics compared to waitlist controls in Calgary, Alberta FPs in Ontario treating CNCP	Patients from a pain clinic waitlist were randomly assigned to usual care or standard telephone consultations, with outcome measures including NRS pain intensity, the Pain Disability Index (PDI), the Short Form (SF)-36 Health Survey, Patient Global Impression of Change scale, Pain Treatment Satisfaction Scale, a PCP satisfaction questionnaire, and a knowledge transfer questionnaire Semi-structured interviews for determining perspectives on opioid prescribing and the barriers/enablers to prescribing opioids for pain	No significant differences were observed between the 2 groups in NRS scores, SF-36, PDI, and Patient Global Perception of Change
			Knowledge transfer related to evidence-based practice of PCPs in the intervention group was between 63.1% and 91.6%
			PCPs reported the telephone consultation was convenient, timely for patient care, an efficient use of time and a useful knowledge transfer strategy
			Beliefs about consequences and capabilities, behavioural regulation and professional role and identity were influential determinants on whether or not a PCP will prescribe opioids
			Most PCPs felt they lacked appropriate answers to assist patients in managing their pain
			Incongruency between usage of opioid guidelines and knowledge of non-pharmacological therapies
			Better communication required among health professionals and with patients about opioids
Desveaux 2019; Qualitative study; 22 participants	FPs in Ontario treating CNCP and prescribing opioids	Semi-structured interviews for determining barriers/challenges in prescribing opioids, surrounding beliefs, and support sources	Discrepancy between PCP training & current practice, tension between PCP & patient expectations
			Influence of length of time in practice and strength of therapeutic relationships on opioid prescribing
			Interventions to address these discrepancies must include changing PCP’s beliefs in their confidence prescribing opioids
			FPs need more support through tailored educational strategies, and shared decision-making with allied healthcare professionals
Furlan 2018; Pre-post intervention study; 170 multidisciplinary participants	ECHO (Ontario Chronic Pain/Opioid Stewardship) program for managing chronic pain in primary care	ECHO model as described above in Carlin 2017	ECHO is a useful template for creating other education programs
			96% of participants were satisfied with ECHO as a learning experience
			Significant increase in self-efficacy
			Significant increase in knowledge
			Enabled best practice dissemination, reduced variations in care, enhanced satisfaction in providing care, improved quality of care, and reduced professional isolation
Furlan 2020; Cross-sectional survey; 265 family physician respondents	Canadian FPs’ management of CNCP and opioid prescribing post-2017 guidelines, update to Allen 2013 survey	Survey assessing self-reported practices of opioid prescribing and knowledge, barriers, and facilitators to adhering to the 2017 opioid prescribing guideline	Wait times to access pain specialist exceeded 1 year in 24% of respondents, and 23% had no access at all
			Knowledge assessment of opioid use revealed physicians averaged 55% correct responses
			Concerns about long-term adverse effects and lack of evidence for use in CNCP major reasons for not prescribing opioids or not using strong opioids
			An average of 66% of respondents adhere to recommended practices while monitoring patients on opioids
			Enablers to opioid prescribing were access to an opioid prescription history from provincial monitoring programs and access to pain/addiction specialists, followed by access to nonpharmacological options
Goodwin 2018; Qualitative survey; Unknown number of participants	Family physicians in Nova Scotia	Semi-structured interviews to determine opioid prescribing patterns, core issues and challenges with respect to opioid prescribing, and what kinds of supports would be helpful	Challenges in prescribing opioids entailed:
			Complexity of CNCP management, physician-patient relationships, concern for diversion, lack of training, and systemic issues (such as wait lists for specialists and cost of pain medications)
			Beneficial supports included:
			Education on CNCP management, prescriber tools such as opioid agreements, and prescriber guidelines
Hassan 2020; Focused group discussion; 20 attendees (5 family MDs, 5 NPs, 1 PA)	Project ECHO workshop participants in Ontario	Focused group discussions on PCPs’ attitudes toward interprofessional care (IPC) and interprofessional education (IPE) on managing patients with CNCP and the impact of ECHO on these attitudes	Physicians viewed IPC/IPE as important for learning aspects of other professionals’ skills
			Negative aspect entailed a difference in communication practices (ex. case presentations) which was felt to slow the progress of time-limited ECHO sessions
			Physicians valued input from non-physician experts in the ECHO hub, with notable learning opportunities
			Participants appreciated belonging to a learning community of practice, even though remote
			Non-physicians realized necessity of IPC/IPE in managing patients with CNCP, found it a good means of acquiring new knowledge around pain management, and for changing interprofessional communication in their practice, while simultaneously providing a venue to have their voices heard
Julien 2015; Cross-sectional survey; 93 FPs	FPs in Northern Quebec	Surveys were sent with an adapted Dillman Total Design Method to assess current and desired knowledge of CNCP and its treatment	Respondents estimated that 25.9% of their patients had CNCP
			Most had taken <10% CME activities involving CNCP in the previous year
			On average, participants perceived a gap between their current knowledge level of CNCP management and desired knowledge level of 28%
Kaasalainen 2007; Cross-sectional survey; 16 NPs	NPs providing primary care in long-term care facilities in Ontario	Survey assessing 33 activities related to pain management, whether they currently performed or should perform said activities, and barriers to fulfilling these roles	81.3% used pain-assessment tools, less than half use clinical practice guidelines
			NPs reported less involvement in prescribing and adjusting pain medications, providing leadership in pain management, or engaging in pain research despite most feeling they should be more involved
			Barriers identified included time constraints, prescribing restrictions, lack of knowledge, difficulties assessing pain (especially in dementia patients), poor collaboration with physicians, and various parties’ reservations about the use of opioids
Lakha 2011; Cross-sectional survey; 148 participants	Tertiary pain clinic referrals made by FPs in Toronto, Ontario	Determining what prompts and limits PCPs from referring their patient with CNCP to a pain clinic	Reasons for referrals included requests for nerve blocks, desire for expertise of the program, concerns about opioid prescriptions, “last resort” for complex patients, medicolegal concerns, or patient request
			Barriers to pain clinic referrals are long wait lists, patient preferences, distance from clinic, language barriers, perceived futility, inability to provide follow-up, or concerns about being prescribed opioids
			Need to promote prompts for PCP to refer to pain clinic
Lalonde 2014; Cross-sectional survey; 137 physicians and 110 pharmacists	PCPs in Laval, Quebec	Participants were sent the KnowPain-50 Questionnaire to assess knowledge attitudes, and beliefs of physicians regarding pain and its treatment, sociodemographic information and previous training, and needs and preferences for a continuing education program	Overall unadjusted mean scores for physicians was 69.3%
			Highest scoring areas included implementation of a treatment plan and general notions of pharmacotherapy and management of analgesic side effects
			Scores were lower in specific patient populations (elderly, those at risk of addiction), specific types of pain (e.g. neuropathic), initial pain assessment, defining goals and expectations, treatment plan development, reassessment and management of longitudinal care, as well as legislative issues
			Physicians acknowledged need for continuing education citing most relevant need for knowledge on differential diagnoses of chronic pain syndromes (71.3%); >40% reported interest in injection-type intervention techniques and indications and referral procedures for pain clinics; 1/3 want training on pain assessment and on physical and psychological follow-up for CNCP patients
			Physicians cited problem-based learning as preferred format, and 1 in 3 would appreciate a day at a pain clinic
Lalonde 2015; Qualitative survey; 6 PCPs (53 multidisciplinary and patient participants)	1-day workshop in Laval, Quebec	Focus and nominal groups to determine challenges and priority interventions in CNCP management and to review current management strategies in primary care, aspects of the chronic care model, and interventions of change	Participants noted knowledge gaps, working in silos, lack of awareness that CNCP is an important clinical problem, difficulties accessing health professionals and services, and lack of patient empowerment
			Suggested interventions include promoting patients’ active participation in their treatment through patient education and log-books/webpages, interdisciplinary approaches, identifying regional experts and training them to disseminate information, defining care paths from primary, secondary, and tertiary care to optimize access to specialized resources, and creating guidelines
Liddy 2016; Cross-sectional study; 93 PCPs (86 family physicians and 7 NPs) Maclean 2017; Qualitative study; 2 primary care physicians Midmer 2006; RCT; 88 participants	Review of all eConsult cases submitted to chronic pain specialists in the Champlain LHIN (Ottawa and surrounding area) Interviews with rural physicians in Newfoundland Online continuing medical education event for FPs on opioid- and benzodiazepine-prescribing for CNCP	Usage data and provider responses to a mandatory closeout survey were analyzed to determine response times, case outcomes, and provider satisfaction Semi-structure interviews focused on barriers to deprescribing opioids and managing CNCP in a rural setting All participants completed a workshop and the intervention group then completed 10 weeks of weekly email case discussion	Median response time to PCP questions by specialists was 1.9 days, and in 74% of cases PCPs received advice suggesting a new /additional course of action
			PCPs were able to avoid referring patients for a face-to-face specialist visit originally planned in 36% of cases and only 44% of eConsult cases resulted in a patient referral
			90% of PCPs rated the service 4 or 5/5 for overall value for patients, and 92% rated it 4 or 5/5 for value to providers
			Study underscores trend in eConsult ability to deliver prompt access to specialist care, possibly reduce pain clinic wait times, and improve patient safety
			Barriers identified include system-related barriers such as addiction treatment and pain clinics, lack of training in pain management and opioid deprescription, lack of interdisciplinary teams
			Web-based learning through email case discussions were helpful, inexpensive and an informal way of continuing medical education albeit most likely helpful in specific and concrete clinical behaviours
Petrella 2006; Pre-post intervention study; 659 participants	Joint Adventures program (1-day workshop) offered in 9 out of 10 Canadian provinces	The combination of script concordance (similar to PBL) + continuing medical education program were used to assist family doctors in acquiring knowledge, skills and tools to improve their management of musculoskeletal disorders	Pre-test found knowledge gaps between level of information participants had before the workshop compared to the level they would like to have
			Specific knowledge items included: differentiation of pain source from inflammation vs. mechanical pain, diagnostic tests, treatment, risk with NSAIDs, exacerbation of pain, and how to utilize community resources
			Qualitative responses identified that changes to clinical practice included more use of complementary therapy, avoidance of NSAIDs, better history and physical exams, and more aggressive pain management
			High degree of satisfaction among participants
Poulin 2018; Quality improvement initiative using a cross-sectional design; 194 PCPs Rice 2018 Qualitative study; 13 medical students and residents	PCPs referring patients to a tertiary pain clinic in Ottawa Interviews with medical students and residents in Toronto, Ontario	Pain consultation requests reviewed for wait-listed patients appropriate for electronic consultation were contacted for consideration of eConsult and a cross-sectional survey was used to analyse outcomes Interviews focused on their experiences treating patients with chronic pain	26% indicated they would use eConsult, 8% indicated their patient could be removed from the clinic waitlist
			Of patients that physicians indication they would use eConsult for 35% subsequently had an eConsult submitted
			Subsequent monthly volumes of eConsult submissions increased
			39% of reviewed PCP referral questions could be at least partially addressed through electronic consulation and may in future reduce wait times for tertiary pain care
			Physicians’ opinion about patients with chronic pain increasingly negative, leading to a decline in empathy
			Subjective nature of chronic pain makes it difficult to evaluate and doctors feel frustrated because of their inability to cure chronic pain (doctors feel defeated by these patients)
			Chronic pain patients lack educational value (preceptors shielded their students from exposure to these kinds of patients during training)
			Medical students need the opportunity to reflect on the skills that are required to provide patient-centred care for this population
Roy 2017; Cross-sectional online survey; 1092 physicians (653 FPs)	An online survey of physicians in Quebec in 2015	Insight on FPs’ concerns, practices, and needs with respect to the management of CNCP	FPs rated their academic training on pain as poor and expressed a desire to have a continuing education program to feel more confident treating CNCP
			Training needs identified included patient evaluation and differential diagnoses of chronic pain syndromes, alternatives to opioid management, monitoring pain, and evaluating and managing risks of abuse, misuse, tolerance, dependence, and diversion
			Access to free telephone or online consultation service with a medical expect was considered useful as reinforcement and as a supportive measure
			Prescription monitoring programs were deemed important for patients who use opioids
Squire 2009; Cross-sectional survey; 20 PCPs	PCPs in BC surveyed by questionnaire	Physicians participated in a 3-month pilot project in chronic pain management involving a one-day symposium, online learning, discussion/case presentation, blog and optional mentorship	Physicians rated the symposium most effective and the online learning least effective program component due to time constraints
			Top-ranked topics included assessment tools and sensory evaluation of neuropathic pain
			Time-sensitive pain assessment and outcome measurement tools affected most practice change
Webster 2019; Qualitative study; 19 primary care clinicians and 8 nurses	PCPs in urban, rural, and Northern settings in Ontario	Interviews with physicians focused on the question “How do PCPs describe the work they do in caring for patients with complex chronic conditions?” for 30-90 minutes for a total of 61 formal, semi-structured interviews, along with 40 hours of observational data in clinical settings by shadowing PCPs daily work in caring for complex patients	Themes identified include:
			dealing with substantial socioeconomic status disadvantages
			disjuncture between patients’ hopes and expectations for pain management and the reality of what physicians can provide (especially in current pressures to restrict opioid prescriptions)
			saying no to patients
			difficulties testing, measuring or accurately evaluating pain in absence of clear biomarkers
			limited access to non-pharmacological health services/supports
			difficulties engaging patients in lifestyle modification
Webster 2019; Qualitative study; 51 PCPs	PCPs in urban rural, and Northern settings in Ontario	Interviews examined patient complexity from PCPs viewpoint and asks about the utility of defining patient complexity as multimorbidity	Findings included: poverty, mental health issues, and history of trauma rendered patients more complex interventions needed by such patients exceeds the scope of medical expertise and social issues complicate otherwise potentially straightforward medical problems, especially in chronic pain patients
Weinberg 2010; Cross-sectional survey; 190 PCPs Wingert 2020; Cross-sectional survey; unknown number of participants	Surveys completed at CME events in Canada Surveys sent by email to practicing physicians, NPs, and residents in Saskatchewan	Multiple choice questions regarding prevalence of chronic pain, screening for opioid misuse risk, acetaminophen dosing, risk factors for NSAID-induced GI events, and codeine metabolism Survey with questions to evaluated burden of CNCP to providers, identify competencies/deficiencies, barriers to care, and resources needed to improve CNCP in Saskatchewan	Majority of respondents not aware of chronic pain prevalence
			Lack of awareness on adverse effects of various medications commonly used in primary care
			Only 18.3% use validated opioid misuse risk tools to screen and 20.3% do not screen patients at all for opioid risk
			Confidence with managing opioid rotation and tapering was 51.7% and 65.3% respectively
			Confidence with managing primary and secondary pain conditions were 60.5% and 75.5%
			Barriers identified include financial barriers, lack of time for patient education, and lack of access to a pain specialist or interdisciplinary pain specialists

CNCP: Chronic Non-Cancer Pain; ECHO: Extension for Community Healthcare Outcomes; NSAIDs: Non-steroidal Anti-inflammatory Drugs; FPs: Family Physicians; LHIN: Local Health Integration Network; PCP: Primary Care Physicians; PBL: Problem-based Learning; RCT: Randomized Controlled Trials; WSIB: Workplace Safety and Insurance Board.

### Stage 5: Collating, Summarizing and Reporting the Results—Qualitative Analysis

We adopted Braun and Clarke’s thematic analysis for this scoping review.^37^ Specifically, we performed a reflexive, semantic, inductive, and deductive approach, which will be explained herein. The authors remained reflexive of their social and diverse professional locations and how these backgrounds informed the research project, from conception to manuscript preparation. Semantic (the meaning in language) relationships between included articles’ concepts were explored. Data were coded inductively without an a priori conceptual framework, in keeping with our research aims to provide an overview of PCPs in managing CNCP and to identify gaps in the literature.^37^ We subsequently deductively coded the data using the CanMEDS framework for discussion purposes and as a means by which to inform future education program development.

An iterative step-by-step thematic analysis was performed by two of the authors (V.M. and M.E.), where the included articles were first read in full in order to find patterns, followed by open coding of the data. Braun and Clarke defined a *code* as a “feature of the data (semantic content or latent) that appears interesting to the analyst and refers to ‘the most basic segment, or element, of the raw data or information that can be assessed in a meaningful way regarding the phenomenon.’”^38^ These codes were subsequently grouped into categories, based on conceptual similarities, followed by grouping into emerging themes.^37^ An iterative approach ensued where each reviewer subsequently ensured that each theme was coherent and representative of all of the data.^36^ Subsequently, the two authors compared emerging themes and came to consensus on a common coding framework.^37^ The common coding framework was subsequently applied to all articles included within this scoping review.^37^

For credibility, confirmability, and dependability,^37,39^ the research team triangulated all independently coded emerging codes, subthemes, and themes to create the final coding book, and the senior author reviewed all codes, subthemes, and emerging themes, ensuring accuracy and consistency between the included manuscripts and the available literature.^40^ The researchers also took memos throughout the research cycle to document reflections around data inclusion and analysis. Coding was iterative in nature, meaning that any newly emerging codes and themes were reviewed against all included articles, ensuring that data extraction was complete.

## Results

### Search Results

**Figure 2.**
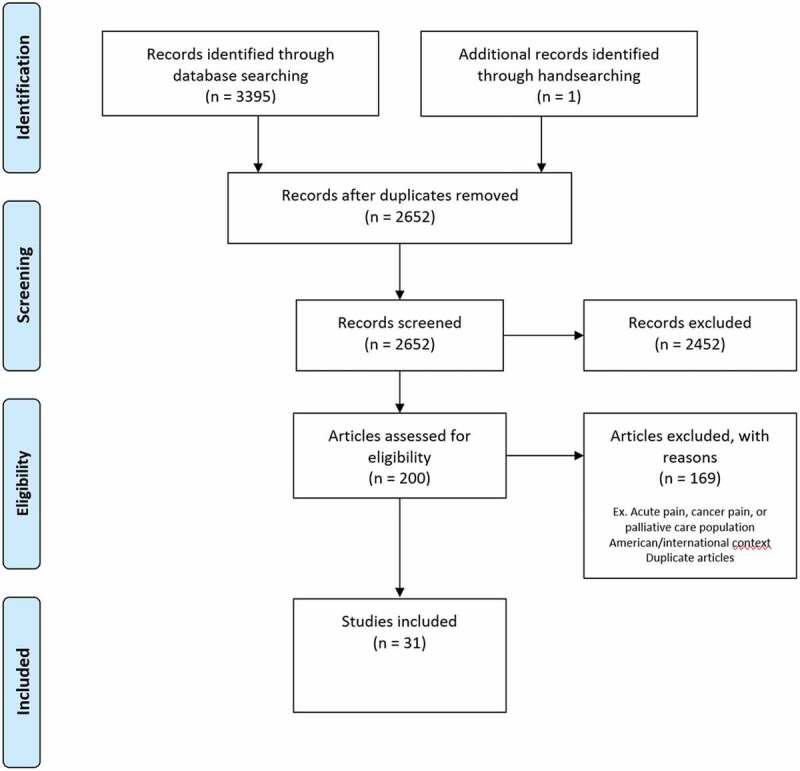
Preferred Reporting Items for Systematic Reviews and Meta-Analyses flow diagram of study selection process.

Our search strategy initially identified 3395 articles, and 743 of these articles were removed as duplicates. Another 2452 articles were excluded because of lack of relevance, and the full texts of the remaining 200 articles were assessed for eligibility based on the inclusion criteria previously outlined. Thirty-one of these articles, published between January 2006 and May 2021, met inclusion criteria for this scoping review^15–17,20,24,41–66^ (Figure 2). It became apparent during initial article screening that some studies would have met the aforementioned inclusion criteria from the era of high-dose opioid prescribing in the 2000s, in which the medical literature at that time was critical of physicians hesitant to prescribe higher “therapeutic doses.” Education suggestions for PCPs from such studies often emphasized increasing opioids for CNCP beyond what would be accepted in current guidelines as best practice. The authors therefore decided that, in the present-day context, such studies would not contribute useful information to this scoping review. Three such studies were identified and excluded.^67–69^

### Characteristics of Included Studies

A summary description of studies included in this review is presented in Table 1. All of these studies represent Canadian PCPs surveyed or interviewed either in in-person workshops, in tele-conferences, by mail, or through online platforms. Of the 31 included studies, 10 (32%) used a qualitative study design,^15,16,20,45,46,52,54,57,60,64^ 16 (52%) used a quantitative design (one randomized controlled trial,^48^ one randomized experimental design,^65^ 14 cross-sectional surveys,^17,24,42,43,47–49,53,55,56,59,61–63^), and 5 (16%) studies used mixed methods.^41,44,51,58,66^ All studies described the methods used to obtain data, and all but two studies^46,62^ provided the number of participants. The number of PCP participants in these studies ranged from 6 to 710, for a total of 4389 PCPs out of 5063 participants. A subanalysis of included studies found that four of the included studies were penned by authors from Québec and focused exclusively on PCPs within Québec, with Quebec participants totaling 889, or 20% of the total PCPs captured by this scoping review.^16,49,56,61^ Additional difficulty in quantifying numbers of participants from Québec may have been captured in the 9 studies targeting PCPs across Canada, of which only 2 studies reported actual numbers of participants from Québec, capturing a further minimum of 77 PCPs from Québec, with likely additional unreported participants from Québec in the other pan-Canadian studies.^44,59^

All included studies explored PCPs’ perspectives on managing chronic pain. Eighteen articles focused exclusively on physicians’ perceptions,^15,24,42–46,48,50,51,53,54,56,59,61,64–66^ and one article focused exclusively on nurse practitioners’ perspectives.^47^ The remaining articles discussed perspectives of family physicians, while also including nurse practitioners^20,41,60,62,63^ and allied health care workers including physician assistants,^20,41,60^ nurses,^16,20,22,41,54,60^ pharmacists,^16,20,41,49,58,60^ dentists,^58^ social workers,^20,41,60^ physiotherapists,^16,20,41,60^ occupational therapists,^20,41,60^ psychologists,^16,41^ chiropractors,^41^ kinesiologists,^41^ and dietitians.^41^ The PCPs in the studies ranged from recently graduated physicians and NPs to those with up to 49 years of experience.^41^ Most geographical regions of Canada were represented, with nine studies surveying physicians all across Canada^17,24,42,44,51,59^; other studies included participants from specific provinces: Newfoundland and Labrador,^64^ Nova Scotia,^16,46^ British Columbia,^53^ Alberta,^65^ Saskatchewan,^62^ Quebec,^16,49,56,61^ and Ontario.^15,20,41,43,45,47,48,50,52,54,57,60,63,66^

Methods of evaluation for the 21 quantitative and mixed methods studies included a questionnaire or a survey to obtain participant responses.^10,17,24,41–44,47–51,53,55,56,58,59,61–63,66^ The mixed methods studies allowed for open-ended responses on the questionnaires.^43,44,47,51,59^ The study implementing a randomized controlled trial design with both groups completing a workshop and the intervention group receiving an additional 10 weeks of e-mail case discussions also used a blinded assessor postintervention to assess participants’ knowledge following the intervention.^51^ Of the 10 studies employing a qualitative design, 5 used semistructured interviews,^15,45,46,54,64^ 3 used focus group interviews,^16,20,60^ and 2 used an open-ended interview style.^52,57^

### Unifying Themes in Studies

Eight common themes were identified and grouped under the following four overarching themes: pain medicine competencies and practice, interprofessional collaboration, attitudes and therapeutic relationship skills, and strategies to reduce gaps in care (Table 2).^48,52,55,48,52,55,58^

**Table 2.** Scoping review inductive coding and emerging themes.

Categories	Subthemes	Themes
Inconsistency/limited experience in applying validated instruments for assessing pain	Improving pain assessment and appropriate pharmacological management	Pain medicine competencies and practices
Difficulty in assigning differential diagnoses for etiology of pain		
Uncertainty about choosing first-/second-/third-line analgesics		
Transition from pro-opioid CNCP management to deprescribing with new opioid guidelines	Advancing opioid prescribing and deprescribing strategies	
Opioid prescribing skills, screening, and attitudes toward prescribing		
Limited use of allied health such as physio- and psychotherapies in managing pain	Nonpharmacological interventions to manage pain	
Absence of multidisciplinary care leading to professional isolation in managing pain	Challenges in building interprofessional collaborations	Interprofessional collaboration
Impact of exposure to interprofessional education on improving multidisciplinary care		
Lack of clearly defined referral and care paths	Deficits in local and regional resources	
Long wait times to access resources for care		
Physicians find it difficult to trust patients’ pain reports when opioids are prescribed	Therapeutic relations between PCPs and patients with CNCP	Attitudes and therapeutic relationships
Stress on PCPs in managing mental health and complex social needs of patients with CNCP		
Difficulties with time management when treating patients with CNCP		
Lack of exposure to CNCP treatment options in medical and nursing school curricula	Deficits in training PCP trainees in managing CNCP	
Absence of comprehensive training programs for managing CNCP		
Continuing education via tele-mentoring (ECHO), online modules, workshops/conferences		Strategies to reduce gaps in care
Benefits of telephone/virtual consultation services for care of patients with complex CNCP		
Systemic improvements to streamline access to tertiary pain care centers		

### Theme I: Pain Medicine Competencies and Practices

## Improving Pain Assessment and Appropriate Pharmacological Management

In general, both in surveys and with in-depth interviews, PCPs reported challenges with assessing pain^49,53^ and navigating modalities of treatment for CNCP.^15,45,49,51,62,64^ PCPs described struggling with assessing and quantifying patients’ pain in the absence of clear biomarkers or imaging findings supporting a diagnosis of pain.^15,49,54,57^ Multiple studies included in this review found that validated scales to measure the intensity of pain were not universally employed.^15,16,24,42,49^ PCPs also reported difficulty with proposing differential diagnoses for chronic pain syndromes.^56^ However, PCPs acknowledged the importance of recognizing and treating common comorbidities associated with chronic pain, such as anxiety and depression.^15,16,42,49,54,56^

Standardized assessment tools and clinical practice guidelines, along with staff education and support for implementation, were considered helpful by NPs in managing pain^16,47^; however, only 50% of NPs reported using clinical practice guidelines developed by other non–nurse practitioner health professional groups.^47^ Despite awareness of existing tools and guidelines, many PCPs revealed in a semistructured interview that they had limited functional knowledge of or practice in using them.^16^ An exception to this observation was awareness of opioid prescribing guidelines, which surveyed PCPs commonly reported implementing in their practice.^42,59^

Across studies, PCPs also reported uncertainty in executing a stepwise approach to pain management in choosing appropriate pain medications to prescribe to patients,^20,44,57^ including determining when opioids were appropriate.^16,20,44,49,50,56,57^ One survey assessed PCPs’ pharmacological knowledge in pain management and found that many clinicians were not aware of adverse effects of commonly used analgesics.^17^ However, other surveys found that PCPs had concerns around overmedicating patients and adverse effects of medications, particularly in elderly patients and patients with cognitive impairments.^47,49,56^

## Advancing Opioid Prescribing Practices, Including Deprescribing Strategies

### Transitions from pro-opioid CNCP Management Toward Deprescribing

The theme dominating PCPs’ practice challenges revolved around managing opioids for patients with CNCP. PCPs interviewed in two qualitative studies reported feeling unsupported in the transition from a time when opioids were recommended for treating CNCP to the current opioid crisis and guidelines recommending de-escalation of prescribed doses of opioids.^15,57^

Lack of knowledge and resources for opioid prescribing has translated into gaps in implementation of new guidelines and created new barriers in treating CNCP effectively. Some PCPs used a strict approach to opioid prescribing, including regimented use of opioid contracts and requiring objective evidence of pain as an indication to prescribe opioids, which was reported to complicate the provider–patient relationship^15,45,46,50,56,58^ Despite guidelines supporting the practice of tapering opioids in patients who fail to achieve pain management goals with high doses of opioids, PCPs often did not take this approach,^15,55^ though some surveyed PCPs interpreted guidelines as mandating opioid tapering.*^59^* PCPs in several qualitative studies described challenges in deprescribing opioids in patients on high doses because patients often resisted this and expressed fear of exacerbation of their pain,^15,20,57,59^ and prescribers reported lack of knowledge in opioid deprescribing.^62,64^ PCPs interviewed also reported lacking appropriate knowledge and skill resources to assist patients in managing withdrawal symptoms during opioid tapers, often leading patients to return to previous high doses.^15^ Many studies reported that being aware of the risks of opioid misuse increased PCPs’ hesitancy in prescribing them^15,44,45,48,55,57^; studies indicated that PCPs wanted more education on risks and benefits of various opioids,^15,20,49,55,57^ and use of long-acting opioids elicited concerns from PCPs in one qualitative study about the risk of addiction despite lack of evidence that long-acting opioids increased risk of addiction compared to short-acting opioids.^45^ PCPs also expressed concerns that some patients with CNCP tend to exaggerate pain in order to obtain opioids.^16,49,52,57^ Conversely, PCPs interviewed were more comfortable prescribing opioids when patients expressed anxiety or avoidant behavior with respect to starting opioids.^15^ One PCP reported that “knowing the patient” strengthened their confidence that opioids may optimize function based on the patient’s beliefs around opioids, mental health comorbidities, and addiction risk.^15^

There were some differences between the approach to prescribing opioids for less- and more-experienced PCPs. More-experienced PCPs reported recognizing their patients’ concerns and behaviors about opioids to reduce enforcement measures in opioid prescribing.^15,45^ Less-experienced PCPs reported more frequent use of the 2017 Canadian Guideline for Opioids for CNCP than more experienced PCPs,^15,45,57^ exercising more caution initiating opioids, and expressed fear of disciplinary action from professional regulatory bodies for inappropriate prescribing.^15,20,55,57^ Several studies reported criticism of the Canadian guidelines for opioids due to the absence of strong evidence, clear and actionable strategies, and the unavailability of the recommended resources to support avoidance or the use of lower doses of opioids.^15,45,57,59^

#### Screening for Opioid Misuse

Surveys found that only 20% PCPs used validated tools to screen for the risk of opioid misuse prior to initiating opioids,^17,56^ and another 20% did not routinely screen their patients’ opioid regimes.^17^ A survey from 2018 documented an increased trend in the use of opioid risk tools and screening as compared to 8 years prior.^42^ Further, in four studies, monitoring with surveillance measures, such as urine drug screening, was deemed stressful by PCPs and was reported to create tension between the PCP and the patient.^15,20,45,56^ Monitoring opioid effectiveness by assessing for functional improvement in patients with CNCP was acknowledged by PCPs as an important factor in guiding opioid prescribing,^44,55^ and in three studies, PCPs acknowledged that opioid prescriptions should be connected to goals of functional improvement beyond pain control.^24,43,57^ PCPs also agreed that clear treatment goals should be established with patients prior to initiating opioids for treating CNCP.^44,49^

#### Attitudes and Perceptions Toward Opioid Prescribing and CNCP Management

In qualitative interviews, some PCPs questioned whether CNCP management, including opioid prescription, should be their responsibility,^45^ and other PCPs expressed the realistic concern that patients may be denied adequate treatment if PCPs do not manage their patients’ CNCP.^15,20^ A common reason for referral to pain clinics was PCPs’ concerns about opioids; however, some PCPs reported frustration with pain clinics that at times prescribe high doses of opioids for CNCP that PCPs did not feel comfortable managing or continuing to prescribe.^48^ PCPs also reported that patients were often initiated on opioids by other prescribers such as surgeons or pain specialists, with instructions to follow up with their PCP, creating difficulty for PCPs when deciding whether to refill patients’ requests for opioids.^15,45,54,55^ This was perceived by the PCPs as offloading responsibility without adequate communication or guidance,^45^ resulting in disorganized care and poor outcomes for patients, such as dependency, illegal drug use, or unintentional withdrawal.^15^

## Nonpharmacological Interventions to Manage Pain

PCPs primarily treated patients with pharmacological therapies such as opioids, antidepressants, and anticonvulsants, whereas the use of nonpharmacological treatments such as engaging allied health colleagues was low in surveys, even if patients were able to fund these treatments.^24,44^ PCPs expressed interest in learning about the role of nonpharmacological pain treatment opportunities, such as cognitive behavior therapy, to manage CNCP.^15,42,44,49,59^ One survey found that the most frequent nonpharmacological treatment modality utilized by PCPs for treating CNCP was psychotherapy, with 20% of PCPs exercising this option.^24^ Referrals for consultations to physiotherapists, chiropractors, and osteopaths were reported by PCPs to improve patient self-management and satisfaction, as well as reduce the need for medications and consultations with PCPs.^16^ However, PCPs felt that their attempts to encourage patients to adopt lifestyle modifications, such as weight reduction, were often met with resistance from patients, who expressed preference for pharmacological therapy in one qualitative survey.^57^ Nevertheless, by empowering patients to be active participants in their own care, PCPs found it feasible to support them through lifestyle changes.^16,57^

Geographical and financial limitations were significant barriers to PCPs prescribing nonpharmacological interventions.^15,20,44,46,57,59,62,64^ PCPs interviewed reported a greater likelihood of prescribing opioids to patients of low socioeconomic status compared to less affordable nonpharmacological interventions, such as physiotherapy, massage, and psychotherapy.^57^

### Theme II: Interprofessional Collaboration

## Challenges in Building Interprofessional Collaborations

PCPs expressed feeling unsupported in their endeavors to manage CNCP patients within the broader health care system,^45,54,62,64^ and this was perceived as impeding guideline-concordant care.^45,54^ The absence of multidisciplinary care in many clinical settings was believed by PCPs in two qualitative studies to be related to the experience of professional isolation.^15,16^ NPs felt that one of the major barriers to effective CNCP management was poor collaboration with physicians, and trusting relationships between NPs and physicians was perceived as a major facilitator by PCPs.^47^ PCPs also reported relying on consultations with other physicians, pharmacists, nurses, and pain specialists to formulate treatment plans for CNCP.^15,47^ PCPs highlighted the need to collaborate with pharmacists in particular to provide effective treatment for patients.^16,47^ Physicians interviewed also endorsed primary care nurses being trained in pain management to support an multidisciplinary approach,^16^ because nursing staff are increasingly relied upon by physicians to assess CNCP and evaluate adverse effects of pain medications.^15^

PCPs interviewed who worked in a multidisciplinary environment were more confident in prescribing practices and CNCP management strategies.^15^ Focus group discussions found that PCPs generally felt interprofessional education sessions were useful learning for managing patients with CNCP,^58,60^ provided opportunities to be better acquainted with their colleagues and respective areas of expertise, and aided the development of a common language on the topic of pain.^16,60^ Project ECHO (Extension for Community Healthcare Outcomes) was quoted as an example of an interdisciplinary collaboration, in which multiple disciplines convene simultaneously for case-based learning, with the goal of “creating a community” of health professionals; this allows for regular interaction and has been found beneficial by PCPs in chronic pain education and management.^20,41,60^ After participating in an interprofessional education workshop, most participants in a study indicated that they involved other health care professionals more frequently in managing CNCP.^20,41,58,60^

## Deficits in Local and Regional Resources

There was a general consensus from PCPs in studies included in this review about the lack of available resources and access to specialists to support effective pain management.^15,42,45,54,59,62,64^ A lack of clearly defined referral and care paths and limited information on available resources such as pain specialists and multidisciplinary pain treatment clinics were identified as possible reasons for these limitations in semifocused interviews.^16,54^ Long wait times, with the median being 6 months^16^ but ranging as long as 3 to 5 years,^15,16,20,46,48,55^ were identified as a barrier to effective care for patients with CNCP. One study found that long wait times negatively affected patients, because they experienced higher pain intensity, functional interference, psychological distress (depression and suicidal ideation, anxiety, anger), and poorer quality of life.^16^ Other barriers to referral to pain clinics included lack of specialized treatments outside of the context of formal pain clinics, distance from patients’ residences to the pain clinics, inability of pain clinics to offer frequent follow-up visits, and initiation of high-dose opioids by pain clinics that PCPs were not comfortable with continuing to prescribe.^48^

One qualitative study suggested that providing good care for patients with complex CNCP would require significant transformation of the health care system.^54^ PCPs opined that challenges posed by low socioeconomic status of patients with CNCP impacted available treatment options.^57,59^ Five studies reported that PCPs felt the cost of medications was a major barrier in delivering effective pain management.^20,43,44,46,55^ Additionally, limited services available outside of major urban centers impacted patients with CNCP in rural areas, who often had more difficulty accessing recommended resources, such as physiotherapy.^20,57^

### Theme III: Attitudes and Therapeutic Relationships

## Therapeutic Relations between PCPs and Patients with CNCP

A common theme emerged in many studies of PCPs feeling challenged by balancing adequate analgesia with opioids against avoiding potential conflicts with patients or complications such as misuse and addiction.^15,20,45,46,54,57,59^ In one qualitative study, PCPs reported struggling with refusal to prescribe opioids to patients.^57^ Interviews with PCPs indicated that they had difficulty creating a trusting relationship with their patients with CNCP because of the difficulty in judging the legitimacy of patients’ requests for opioids.^15,52,54,57^ PCPs interviewed often felt torn between the goal of reducing patients’ suffering secondary to lack of effective treatments and ongoing pressure to avoid opioids.^15,57^

In two qualitative interviews, PCPs described having to engage with patients’ needs outside of CNCP and physical health, including mental health and social issues such as poverty and marginalization; this contributed to PCPs’ loss of job satisfaction, emotional exhaustion, and depersonalization.^54,57^ Some PCPs interviewed found it difficult to trust pain statements from patients with depression or other mental health concerns, and they delegitimized pain complaints in the presence of psychiatric issues.^15,54^

PCPs also identified the term “chronic” in CNCP as discouraging, because it implies the inability to cure the patient’s pain, evoking dismay and annoyance from patients and underscoring CNCP as a “nuisance” condition.^52^ Patients with CNCP frequently missing appointments was “frustrating” to some PCPs, even though PCPs realized that patients with CNCP may be dealing with significant life challenges outside of appointments.^15,54^

One qualitative study found that PCPs had difficulty navigating broader systemic inequalities affecting patients, resulting in dismissive and stigmatizing perceptions.^54^ Further, some PCPs expressed that “patients’ complex social needs are outside the scope of biomedical expertise, and patients with CNCP should not expect PCPs to resolve their problems.”^54^ The work associated with caring for patients taking opioids was termed by interviewees as “babysitting” rather than “caring,” implying a disconnect from a reciprocal therapeutic PCP–patient relationship.^57^ Medical trainees interviewed in another qualitative study reported struggling with the legitimacy of the patient’s experience, because it was difficult to know how to appropriately respond and treat the patient when a patient’s pain cannot be measured and quantified except through the patient’s own narrative.^52^

Patients with CNCP were often viewed by PCPs as passive care consumers rather than active partners in their treatment.^16^ It was suggested in one qualitative study that patients’ empowerment needs were often not adequately addressed, and many patients were simply unaware of self-management strategies available to improve their CNCP.^16^ There was often a discrepancy in PCPs’ perceptions of adequate pain management; one survey indicated that 82% of physicians were satisfied with their patients’ treatment plans; however, almost the same number of patients with CNCP (79%) noted that their ongoing pain was severe enough that it interfered with their function.^43^

Significantly more time is spent with patients with CNCP compared to patients with other ailments,^43,54^ so a commonly reported barrier to effectively managing CNCP was lack of time.^44,47,54,57,59,62^ PCPs reported avoiding opioids due to time required to adequately manage initiation, monitoring, and tapering, and they felt it was a type and volume of work they were unequipped to handle.^15,16,49,55,57^

## Training Exposures and Attitudes toward CNCP for Medical Trainees

Twelve (46%) studies in this review highlighted PCPs reporting a lack of training in medical school or higher training in treating CNCP.^16,20,46–49,51,53,55–57,59,64^ PCPs expressed disappointment in medical training that emphasized curing (over caring for) patients as the cornerstone of good medical practice.^49,52^ They were also concerned that opioids were taught as the gold standard for pain management while a growing opioid-phobic medical culture urges avoidance of opioids altogether for treating CNCP.^15,20,45,57^ Medical students interviewed reported insufficient exposure to patients with CNCP, leading them to believe that such patients were “difficult,” and encounters with these patients were perceived to be challenging unpleasant tasks rather than a learning opportunity.^52^ In this same qualitative study, PCPs reported perceiving previous supervisors and preceptors shielding them from patients with CNCP, giving the impression that such patients had limited educational value.^52^ In one needs assessment survey, fewer than 10% of PCPs surveyed had taken a course involving CNCP in the past year,^61^ and another survey reported that 65.7% of PCPs pursued continuing medical education in other topics.^54^ The absence of a unified training program for PCPs providing a comprehensive approach to treating CNCP was also identified.^16,20,47,48,50,57^ In several qualitative studies, early-career PCPs expressed their interest in learning about skills and strategies necessary for safe opioid prescribing, navigating difficult conversations, and reinforcement of their ability to provide guideline-concordant care.^15,45,57^ However, one of these studies stated that the educational strategies to disseminate guideline recommendations may be inadequate unless they address the interpersonal aspects of patient–provider interactions and PCPs develop skills needed to navigate the multidisciplinary system.^11^ It was noted in two qualitative studies that current medical education curricula do not prepare PCPs to address the pressing needs of patients with CNCP related to low socioeconomic status but rather that the narrative continues to focus on the dangers of opioids themselves, instead of on adverse social conditions leading to widespread exposure to opioids.^54,57^

### Theme IV: Strategies to Reduce Gaps in Care

Models cited as successful in addressing some gaps of care included Project ECHO interactive tele-mentoring,^49,53,56^ interprofessional education workshops,^49,53,56^ online learning modules,^59^ teleconsultations/electronic consultations,^63,65,66^ and streamlining referral pathways with clear tiered approaches to accessing tertiary pain care, such as Quebec’s Pain Centers of Expertise.

PCPs who attended Project ECHO tele-mentoring sessions reported an increase in overall confidence in managing patients with CNCP, citing decreased stress, learning from interprofessional colleagues, and feeling more empathetic toward their patients during clinical encounters.^20,41,60^ ECHO was regarded as an insightful program to learn about responsible opioid prescribing, weaning, and deprescribing.^20,41^ However, PCPs attending workshops on treating CNCP preferred face-to-face interactive learning, including conferences and lectures.^49,53,56^ Workshops highlighting interactive, interdisciplinary, patient-centered continuing education programs were perceived as necessary to fill knowledge gaps, foster mutual acquaintances, develop common discourses among local health care providers, and facilitate appropriate transmission of information among clinicians.^16,20,49,51^ Another survey reported small groups, online learning, or archived videos as preferred learning formats.^59^

Alternative means of knowledge transfer was noted in three studies examining telephone consultations or electronic consultations (e-consults) for specific patients whom PCPs had referred to tertiary pain centers.^63,65,66^ The study that incorporated a randomized control design comparing telephone consultations and usual care found no significant differences in patient outcomes, but PCPs reported satisfaction with the timely feedback on cases and found value in this means of knowledge transfer.^65^ In another study, e-consults were found to be beneficial in suggesting a new or additional course of action in 74% of cases, with a median response time of 1.9 days, demonstrating a means with which pain clinics may have improved waitlists and PCPs receive timely feedback.^63^ In a subsequent quality initiative examining e-consults, patients selected from the tertiary pain center waitlists that were appropriate for e-consults (i.e., did not request interventions, did not involve cancer cases or complex regional pain syndrome, or did not require physical examination) were offered e-consults.^66^ Twenty-six percent of PCPs indicated that they were interested in this service, and 39% of PCP referrals were found by pain specialists to have questions that could at least be partially be managed by e-consults, demonstrating a practical means with a potential to reduce waitlists.^66^ PCPs expressed high satisfaction rates with both telephone and e-consults as timely for their patients and an effective means of knowledge transfer.^63,65,66^

Other means of supporting PCPs involves creating efficiencies within the larger health care system. The creation of pain centers of expertise with an integrated and hierarchical continuum of chronic pain services with designated local primary care clinics, designated regional secondary pain clinics, and tertiary centers with multidisciplinary pain treatment centers linked to affiliated rehabilitation centers significantly reduced wait times for patients with CNCP in Quebec.^16^ This model enabled implementation of standardized consultation forms and evidence-based practice guidelines for the treatment of various types of CNCP syndromes, provided PCPs with tools to virtually discuss challenging clinical patient scenarios with pain specialists, and introduced new communication tools.^16^

## Discussion

This scoping review identified and synthesized the available literature in understanding PCPs’ perspectives on gaps in care in treating CNCP, including knowledge, skills, attitudes, and health care systems. Our review found 31 publications with consistent themes that could be mapped under four overarching themes: pain medicine competencies and practices, interprofessional collaboration, attitudes and therapeutic relationships, and strategies to reduce gaps in care. The perceived void of care options following the shift in opioid prescribing expectations was the challenge reported with the highest frequency, followed by the need for knowledge around pain assessment, nonpharmacological management, and interprofessional collaboration. The need for additional regional supports, means with which to effectively manage patient encounters to maximize limited time, and ongoing attitudes challenging therapeutic relationships with patients, beginning in basic medical education and carrying over into clinical practice, were consistently reported by PCPs across multiple studies in various regions of Canada. The secondary objective of this review was also achieved through identifying strategies identified by PCPs as useful in reducing some of these gaps in care, predominantly in knowledge dissemination and improving access to specialty care.

The four themes identified were deductively coded using the CanMEDS framework as a practical means of reorganizing themes and outlining ways in which medical education at different stages, both prior to entering and throughout practice, can address gaps in care identified by PCPs (Table 3). As an educational framework, the overarching goal of CanMEDS is to improve patient care by outlining abilities physicians should acquire to meet the needs of the patients they serve.^70^ This was felt to aptly complement this scoping review in highlighting roles that PCPs and specialist colleagues need to address in medical education to holistically address gaps identified by PCPs in caring for patients with CNCP. The use of educational frameworks as a bridge between continuing health professions education intended to address complex issues and desired outcomes has been well described in other closely related chronic pain initiatives as a means for achieving demonstrable high-quality changes with regard to patient and population outcomes.^71,72^ Though CanMEDS is a framework centered around medical education, it is notable that there are strong parallels to the domains described in the National Nursing Education Framework for nursing education.^71^ It was thus felt to be conceptually transferable.

**Table 3.** Deductive coding for the scoping review using the CANMeds framework.

		Medical expert	Communicator	Collaborator	Leader	Health advocate	Scholar	Professional
Themes	Subthemes							
Pain medicine competencies and practices	Improving pain assessment	Pain assessment, use of validated tools	Creating reliable virtual platforms for discussing challenging scenarios				Further research into effective continuing education delivery models	
	Advancing opioid prescribing and deprescribing strategies	Supporting the transition of practice guidelines, opioid deprescribing						
	Nonpharmacological interventions to manage pain	Increased use of allied health supports						
Interprofessional collaboration	Challenges in building interprofessional collaboration			Tele-mentoring (e.g., ECHO), optimizing use of physio- and psychotherapy				
	Deficits in local and regional resources			Virtual consultation	Developing local/regional resources for care with primary, secondary, and tertiary pain centers of expertise	Streamlining referral pathways	Availability of in-person or virtual training for updates in pain medicine	
Attitudes and therapeutic relationships	Therapeutic relations between PCPs and patients with CNCP		Knowledge transfer and communication between tertiary pain centers and PCPs			Enhancing access to resources for improving mental and social health		Early exposure to CNCP in medical training to shift negative attitudes toward CNCP
	Deficits in training PCP trainees in managing CNCP	Integrating pain medicine in medical and nursing schools’ curricula						
Strategies to reduce gaps in care		Enhancing pain curriculum in both undergraduate and postgraduate programs	Increasing means by which PCPs can obtain supports in addition to traditional consults via e-consult and telephone consultations	Knowledge transfer through effective use of various technology mediums and shared care models	Sharing expertise between pain centers and primary care	Streamlining referral pathways	Continuous reevaluation of strategies trialed moving forward to foster lifelong learning in the profession	Initiatives such as Project ECHO to increase empathy and confidence in caring for patients with CNCP

With regard to pain medicine education, addressing practice issues in pain assessment, opioid prescribing/deprescribing, and other pharmacological/nonpharmacological management was a clear and frequently reported topic in almost all studies.^15–17,20,24,41–50,52–59,62,64^ A stronger pain curriculum in both undergraduate and postgraduate medical education and continuing medical education courses enhancing these skills would also fulfil the CanMEDS role of “medical expert.”

The domain of interprofessional collaboration was present in many of the delivery models reporting high rates of satisfaction, such as Project ECHO^16,41,60^ and Joint Adventures,^51^ and continues to be an underutilized aspect of managing CNCP. Given the issues of limited access to pain specialists and resources, finding ways to weave the skills of many health professionals in treating patients with CNCP is clearly a valuable adjunct to not just improving patient care but also reducing professional isolation.^15,16,41,60^ Innovative knowledge transfer strategies, whether tele-mentoring or use of technology with e-consults, can both foster increased capacity in PCPs and reduce demand on limited tertiary specialty pain clinics.^20,60,63,66^ A shared care model in treating CNCP may also lead to a community of support that enables stronger provider–patient relationships,^16,20,41,47,58,60^ supporting clinicians’ growth in the CanMEDS role of “collaborator.”

Maximizing local and regional resources also falls under the CanMEDS role of “health advocate.” Though a suggested first step is to reduce disparities in specialized pain care access by increasing the capacity of PCPs to manage CNCP more effectively on their own, more work needs to be done by pain experts and health systems in streamlining referral pathways to access tertiary resources, with Quebec providing one such example.^16^ Sharing expertise between pain medicine specialists and PCPs with ongoing support for PCPs and patients from tertiary pain centers is one approach in improving care of a challenging and undertreated chronic pain population and reducing professional isolation for PCPs.^10,41,63,65,66^ This type of initiative also demonstrates the CanMEDS role of “leader” and is worth consideration for adoption in other parts of Canada. Further, though it was not the purpose of this review to speak to challenges of chronic pain management in the international context, because there will certainly be differences attributable to culture, sociopolitical values, economic differences, and variations in medical practice and other factors, international readers may find elements of this review that resonate with some aspects of providing care in their respective countries and thus may find it a useful document to reflect on means with which to address gaps in CNCP care as well.

This also leads to an argument for the growth needed in the role of the CanMEDS role of “communicator,” in which those pain experts providing tertiary care need to provide strong direct communication to PCPs in active shared care of patients with CNCP and present clear plans at time of patient discharge from tertiary care programs. Further, pain experts need to continue to consider ways of reducing prolonged wait times for patients with CNCP, perhaps in part by engaging in the process of educating PCPs in pain medicine, whether through continuing education and mentorship opportunities or on an individual case-by-case basis through virtual consultations with PCPs.^20,41,63,65,66^ This may minimize less complex patient referrals to tertiary centers and increase overall resources for patients with CNCP in general.

This review found that development of therapeutic relationship skills was also desired by PCPs, b ecause the CNCP population continues to be perceived as a more challenging group of patients,^15,20,45,46,52,54,57,59^ and this theme is more relational in nature, perhaps requiring a reflection of values in medical training. Several of the publications included in this review highlighted attitudes PCPs have about patients with CNCP and the need for better training in early medical education to shift some of the negative attitudes that learners are exposed to.^15,52,54,57^ These findings point toward the root causes of reportedly challenging therapeutic relationships with patients with CNCP because of the hidden curriculum in medical education.^52^ Medical learners need to have exposure to patients with CNCP early on in their training to ensure that they develop skills to manage pain, focusing on “care” not “cure,” and to enlighten learners that these patients do not represent something to be avoided but rather a learning opportunity.^49,52^ This baseline expectation of higher standards of behavior with respect for the diverse challenges faced by patients with CNCP care reflects the characteristics of the CanMEDS role of “professional.”

With regard to preferred delivery models for continuing education of CNCP identified in this review, participants reported a preference for interactive face-to-face workshops over online learning,^49,53,56^ with the element of developing a support community for PCPs considered important.^41^ Using multiple ways of learning and continually evaluating the outcomes of their work with dedication to lifelong learning help foster the growth of the CanMEDS role of “scholar.” It would be valuable to consider further scholarly research in this area to expand conceptual thinking, based on models of continuing education, as to why these delivery models are preferred and why they may be more effective in addressing the identified gaps.

## Limitations

There are some limitations of this scoping review. First, we did not conduct an extensive gray literature search outside of Google Scholar, which may have elicited further perspectives useful to informing the research question. Second, limiting our search to publications in the English language might have excluded articles published in French as the other official Canadian language, but we noted on subanalysis that this review includes representation of perspectives from the province of Quebec. Third, we excluded literature exploring patient perspectives on what PCPs need to learn about chronic pain, which arguably is an important view on delivering patient-centered care and could easily be considered as a companion review in the future. Fourth, we excluded articles from our results with a tone that was critical of PCPs from the era of high-dose opioid prescribing, but some may argue that these perspectives may still be informative, even though these may no longer be considered best practice. The lens of the CanMEDS framework used to describe how reported gaps translate into opportunities for improvement in patient care from a medical point of view may not be wholly reflective of the values and abilities some subgroups of PCPs, such as nurse practitioners, identify with and assumes a degree of overlap of care values. Last, the use of a scoping review approach, though providing a broad snapshot of the current literature, sacrifices depth and detail that could potentially be valuable in understanding the minutiae of PCPs challenges with managing CNCP. A systematic review would perhaps be a valuable future step in addressing specific questions that arise as a result of this scoping review.

## Conclusion

In our scoping review of PCPs perspectives on barriers in the provision of CNCP care, we found consistent themes emerging in the Canadian literature. Though many of these themes represent anticipated challenges, this review is important in uniting findings from many PCP voices and advancing advocacy for PCPs within our respective regions, as well as at a national level, as we seek to act on recommendations made by the Canadian Pain Taskforce to address chronic pain. Further research may be useful in developing conceptual thought around continuing medical education models effective for PCPs and for addressing large-scale population health problems such as CNCP.^73^ As patients on high-dose opioids become less commonplace, educational needs will shift, but this review may also be a valuable starting point in further developing undergraduate medical education and continuing education programs. Our results also highlight themes going beyond provider-level knowledge gaps in CNCP care, including shifts needed in attitudes that will optimize patient–provider relationships and systemic issues that currently contribute to challenges experienced both by patients and their PCPs. Actionable initiatives geared toward transforming knowledge, skills, and attitudes can be informed by the issues outlined in this review, which, if combined with system-level changes, can improve outcomes of patients with CNCP in an otherwise underserviced patient population.

This scoping review brings together a cumulative body of literature on Canadian PCP perspectives from coast to coast on various gaps in the provision of CNCP care at a time we collectively seek not just to simply emerge from an opioid crisis but also to move forward with the work proposed by many researchers who came before us in improving the care of patients living with CNCP.

## Appendix A. Search Strategy for MedlineALL

Ovid MEDLINE ALL 1946 to May 11, 2021
